# Identifying phenotypes in OSA patients with an indication for CPAP treatment using clinical data and experienced symptom severity

**DOI:** 10.1186/s41687-025-00915-z

**Published:** 2025-08-18

**Authors:** Marion Teunissen, Pascal Wielders, Catherine Bolman

**Affiliations:** 1 Catharina Hospital, Department of Pulmonary Diseases, Michelangelolaan 2, 5623EJ Eindhoven, The Netherlands; 2 Department of Psychology, Open University of the Netherlands, Valkenburgerweg 177, Heerlen, 6419 AT The Netherlands

**Keywords:** Obstructive sleep apnea (OSA), Patient Reported Apnea Questionnaire (PRAQ), Phenotyping, Fatigue, Snoring severity, Daily activity

## Abstract

**Background:**

Although the group of patients with obstructive sleep apnea (OSA) is very heterogeneous, OSA’s severity is mainly expressed by an apnea–hypopnea index (AHI), which does not correlate well with the experienced symptom severity. As a first step to develop a more personalized approach for treatment, the purpose of the current study was to create, through cluster analysis, meaningful OSA phenotypes linked to the Patient Reported Apnea Questionnaire (PRAQ).

**Methods:**

Through a survey, new OSA patients indicated for continuous positive airway pressure (CPAP) treatment completed the Epworth Sleepiness Scale (ESS) and the PRAQ to rate their experienced symptom severity. Clinical data, such as the AHI and comorbidity, were assessed from the patient file. Cluster analysis has been performed to derive OSA phenotypes.

**Results:**

Based on the AHI, comorbidity and experienced symptom severity data of 151 patients, a two-step cluster analysis revealed five OSA phenotypes: “no comorbidity”, “hypertension”, “high symptom severity”, “low symptom severity” and “unclassified”. The five phenotypes mainly differ in the experienced level of fatigue, partner-observed snoring severity and symptoms related to performing regular daily activities.

**Conclusion:**

Not only the AHI, but also comorbidity and subjective symptoms should be taken into consideration when diagnosing OSA, assessing its severity and in providing a more patient-oriented treatment, including deciding about CPAP treatment. Not the often-used ESS but the modified PRAQ scales provide relevant information to assess experienced symptom severity. In addition, for an improved prognostication, we propose an evaluation of the CPAP treatment effectiveness for the five reported OSA phenotypes.

## Background

Obstructive sleep apnea (OSA) is diagnosed when the number of periods of apnea and hypopnea per hour of sleep (expressed as the apnea–hypopnea index, AHI) is ≥ 15 [1]. However, the AHI alone is not a good measure for OSA severity and no significant relationship was found between the AHI and subjective symptom severity [1–4]. Therefore, additionally, OSA is also diagnosed when 5 ≤ AHI < 15, together with the presence of OSA-related symptoms or comorbidity [1].

OSA has a strong association with cardiovascular disease and hypertension [5–8], atrial fibrillation [9] and type 2 diabetes [5], which may cause weight gain [10] and obesity. The latter is considered a major risk factor for the development and progression of OSA [11]. Gender seems to play a role in having different symptoms. Males snore more often and feel sleepy during the day [12, 13], whereas females seem to present less typical OSA symptoms, such as fatigue, morning headaches, insomnia or feeling depressed [14, 15].

After OSA is diagnosed, patients are often treated with continuous positive airway pressure (CPAP), but the benefit from such a treatment and how the experienced symptom severity changes over time is not always clear. Phenotyping of the OSA patient group, which is commonly established through cluster analysis, could facilitate a patient-oriented prognosis of treatment effectiveness and could help healthcare providers tailor therapies to specific patient needs and improve clinical outcomes.

With phenotyping, the heterogeneous OSA patient group is divided based on specific characteristics that are as similar as possible for one group but different for others, with the aim of personalizing treatment [2, 16]. In this study, phenotyping was achieved through cluster analysis, based on natural groupings within patient data derived from patterns in symptoms, severity, or physiological indicators. As mentioned, this approach allows for more personalized diagnosis and treatment, helping healthcare providers tailor therapies to specific patient needs and improve clinical outcomes. Randerath et al. [4] emphasized an urgent need for the definition of OSA phenotypes based on a multi-component classification system, combining the AHI, symptomatology, the Epworth Sleepiness Scale (ESS), the impact of OSA on the cardiovascular system and metabolism and any associated comorbidity.

### Heterogeneity

Various studies have aimed to determine phenotyping in OSA patients [2, 3, 17, 18]. Ye et al. [2] revealed three groups of patients with AHI ≥ 15: a group with disturbed sleep, a group with minimal symptoms and a group with excessive sleepiness symptoms. More than half of the patients in the first two groups were less likely to present the stereotypical symptoms of “snoring” and “sleepiness”, hence the researchers concluded that OSA can occur without the characteristic symptoms. Comorbidities such as hypertension and cardiovascular risk were most common in the group with minimal symptoms and least common in the group with excessive drowsiness symptoms. The groups did not differ significantly in gender, body mass index (BMI) or AHI. Bailly et al. [17] also analysed patients with AHI > 15 and found six subgroups based on age, gender, symptoms, obesity, comorbidity and intoxication (alcohol and smoking). Saaresranta et al. [18] divided a prospective follow-up cohort into four groups of patients with AHI ≥ 5 and excessive daytime sleepiness or insomnia. In their study, sleepiness occurred in less than half of OSA patients, with the insomniac OSA patients having more comorbidities than the sleepy ones.

Despite these promising results to define OSA phenotypes, the studies used different cluster criteria and have not yet led to an unambiguous OSA phenotyping that can be used for daily hospital practice. The heterogeneity of the patient group with a diversity of experienced symptoms and symptom severity makes it difficult to define clear criteria to start continuous positive airway pressure (CPAP) treatment and to ultimately assess treatment effectiveness. Consequently, it is not trivial to determine upfront which OSA patients benefit from CPAP treatment. The purpose of this study was to achieve a meaningful classification of the CPAP-using OSA patient group, using already available clinical and demographic data supplemented with experienced symptom severity administered with the recently developed Patient Reported Apnea Questionnaire (PRAQ) [19, 20].

## Methods

Newly diagnosed OSA patients in the Department of Pulmonary Diseases of the Catharina Hospital in Eindhoven, the Netherlands, were invited to participate in this study between August 2019 and February 2020. They were asked to complete two questionnaires in order to assess their symptom severity before starting CPAP treatment.

Patients were included if they were scheduled to receive CPAP treatment and if they were able to complete the questionnaires independently. The criteria for CPAP treatment were: AHI ≥ 15; AHI between 5 and 15 together with symptoms; and when a mandibular reposition device was not possible because of the patient’s dental state.

### Measuring instruments

The ESS [21] and the PRAQ [19, 20] were used to assess symptom severity. Patient files were consulted to retrieve the patients’ gender, age, AHI, the presence of hypertension, atrial fibrillation, diabetes type II and obesity (BMI ≥ 30, as described by the National Institutes of Health) and the results of the anamnesis.

The ESS consisted of eight questions and was used to measure daytime sleepiness. The respondents were asked to estimate the chance that they would doze off in various daily situations using four options (“no”; “slight”; “moderate”; “high”) linked to scores of 0, 1, 2 and 3, respectively. With a maximum ESS score of 24, a score of > 10 is taken as a measure of excessive daytime sleepiness [22].

The experienced symptom severity was registered on a seven-point Likert scale using the original Dutch version of the PRAQ [19, 20]. The original Dutch PRAQ consisted of 33 questions divided into the following six scales: “Sleepiness”, “Fatigue”, “Daily Activities”, “Emotions”, “Symptoms at Night” and “Social Interactions”. We tested whether the original subscales of the PRAQ were suitable for our population by performing a reliability analysis, which is detailed in section “Reliability of the PRAQ”.

### Analyses

The data obtained from the questionnaires and the patient files were processed using the Statistical Package for the Social Sciences (SPSS), version 27.

To evaluate gender differences in AHI, BMI, age and subjective symptoms, independent-sample *t*-tests and Pearson’s chi-square tests were performed. The importance of the objective criteria was probed with independent-sample *t*-tests on gender, AHI ≥ 15, BMI ≥ 30, atrial fibrillation, hypertension and diabetes type II.

We had a specific group with newly diagnosed OSA patients who received CPAP treatment. Our group of patients might be different from the group with which the PRAQ was established. It was decided not to define new clusters of questions, e.g. by means of a Principal Component Analysis, because the PRAQ-scales were already defined by Abma et al. [19, 20]. However, the coherence of the questions in each of the PRAQ-scales was tested, for our patient group, with a reliability analysis using Cronbach’s alpha. This resulted in six PRAQ-scales: four original PRAQ-scales, one slightly modified PRAQ-scale and one new PRAQ-scale (see “Reliability of the PRAQ”). Subsequently, the average scores for the six PRAQ-scales were transformed into categorical data. Symptom severity was based on the PRAQ-scales only, as the ESS appeared to be non-relevant for phenotyping (see Results). Symptom severity was assessed on a seven-point scale (1–7), with a score of 4 representing “moderate to severe” and 5 representing “severe” problems. We therefore decided to set the threshold for the symptom severity category to a mean score of 4.5, and labelled the experienced symptom severity as low when the average scale value was less than 4.5 and high otherwise.

For phenotyping, SPSS two-step cluster analysis was performed with the important objective criteria (AHI ≥ 15, hypertension and atrial fibrillation) and symptom severity (based on the PRAQ scales) as categorical variables. In addition, significant differences with α = 0.05 were tested using multivariate analysis of variance (ANOVA), with the PRAQ and ESS scores as dependent variables and phenotype as the fixed factor.

## Results

### Patient group

Prior to their CPAP treatment and after giving informed consent, 151 patients completed the two questionnaires. From the 158 invited patients, seven females (4.4%) declined participation without mentioning a specific reason.

### Reliability of the PRAQ

The reliability of the PRAQ was determined using Cronbach’s alpha. The “Social Interactions” scale had four questions with answer options including “not applicable” or “no answer”, which reduced the number of patients for this scale to 58. The Cronbach’s alpha values for the six PRAQ scales were: 0.89 for “Sleepiness”, 0.90 for “Emotions”, 0.64 for “Symptoms at Night”, 0.93 for “Fatigue”, 0.95 for “Daily Activities” and 0.89 for “Social Interactions”. Because Cronbach’s alpha for “Symptoms at Night” was only 0.64, with corrected item-total correlations between 0.20 and 0.57 and without the possibility of increasing Cronbach’s alpha above 0.7, we excluded this scale from further analysis. Based on the relatively low corrected item-total correlation for the snoring question in the “Social Interactions” scale (0.467) and exclusion of the “Symptoms at Night” scale, it was decided to add a separate “Snoring” scale to the PRAQ with the three snoring questions because it is often reported as an important factor, particularly for male patients [17, 18]. Pearson’s correlation between both questions was high (*r* = 0.555; *p* <.001) and Cronbach’s alpha was also sufficient (0.71). For the “Social Interactions” scale, the question “Did you feel ashamed of others for your sleep apnea or for the treatment of your sleep apnea”, which had the lowest corrected item-total correlation (0.407), was removed. Consequently, Cronbach’s alpha for the “Social Interactions” scale was 0.91 and all the corrected item-total correlations were higher than 0.60.

For further analysis, the original four PRAQ-scales “Sleepiness”, “Emotions”, “Fatique” and “Daily Activities” were used, together with slightly modified PRAQ-scale “Social Interactions” and the new PRAQ-scale “Snoring”.

### Differences between clinical characteristics of male and female patients

The *t*-tests and chi-square tests showed significant differences between male and female patients for AHI, BMI and atrial fibrillation (presented in Table 1). The average BMI was significantly higher for female patients (*p* =.044), but a higher percentage of male patients had AHI ≥ 15 (*p* <.001) and atrial fibrillation (*p* =.047).

The mean symptom severities for each of the PRAQ scales are shown in Table 1, where fatigue scored the highest. Because no significant differences in mean symptom severity ratings were found between male and female patients, together with the small number of female patients in this study (*n* = 35), we decided not to split the group based on gender for the phenotyping.

**Table:**
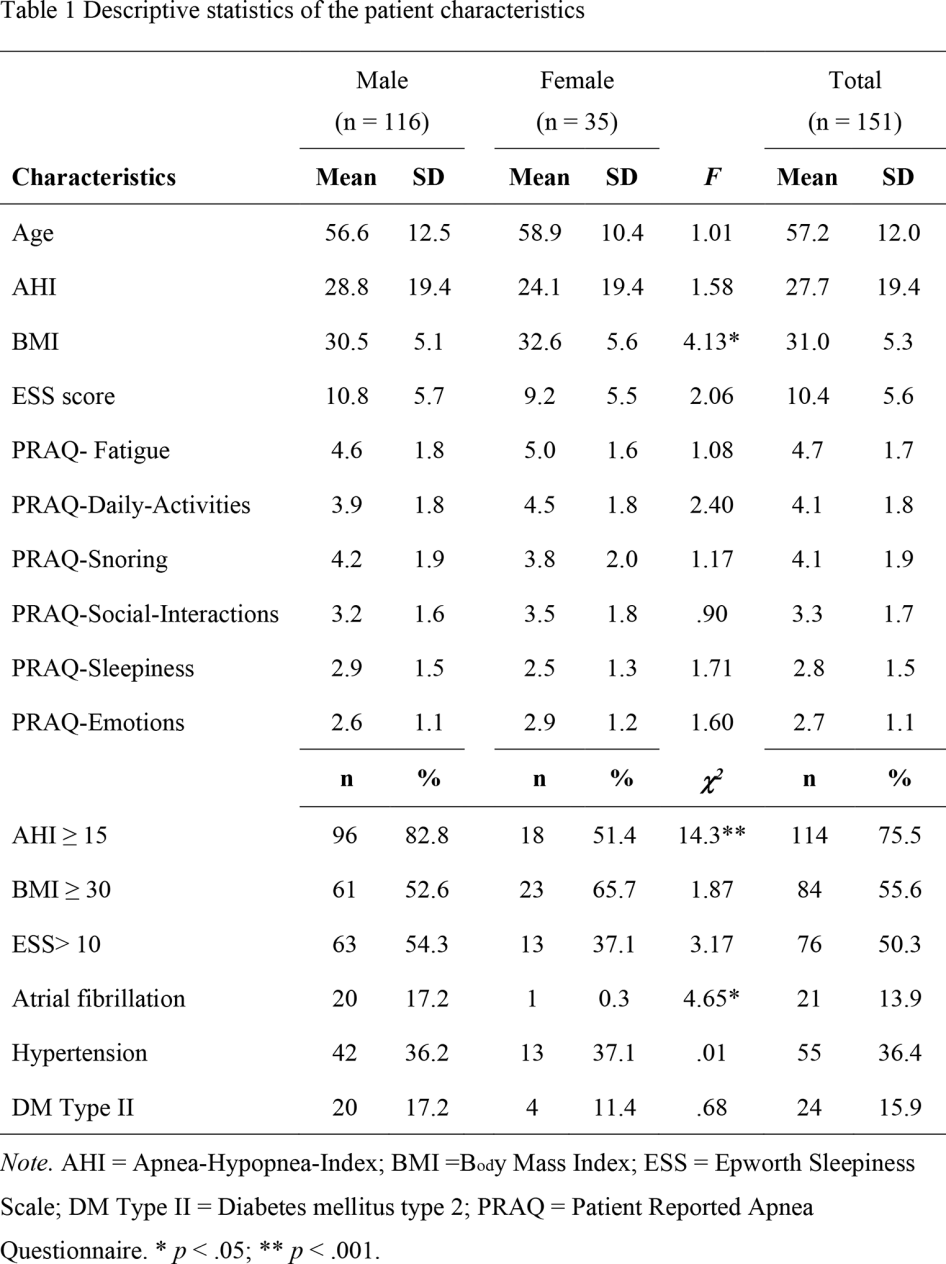

### Differences in symptoms

To determine the impact of the clinical characteristics (AHI ≥ 15, BMI ≥ 30, diabetes type II, hypertension and atrial fibrillation) on the ESS and PRAQ scores, independent-sample *t*-tests were performed. No significant differences were found for patients with and without diabetes type II. The 55 patients with hypertension showed a significantly lower mean for the “Snoring” scale (*p =*.032) than the 96 patients without hypertension. Furthermore, it was found that the mean scores for the “Emotions”, “Fatigue” and “Daily Activities” scales were significantly lower for the 114 patients with AHI ≥ 15 than for those with AHI < 15, with significance levels of *p* =.021, *p* =.039 and *p* =.034, respectively. Finally, the 21 patients with atrial fibrillation had significantly lower mean ratings (*p* <.05) for all the PRAQ scales than patients without atrial fibrillation, except for the “Sleepiness” scale.

The commonly used ESS was not significant in any *t*-test and nor was the PRAQ “Sleepiness” scale. This was not surprising because the ESS and “Sleepiness” were strongly correlated, Pearson correlation *r*(150) = 0.709; *p* <.001. Therefore, only “Sleepiness” was used for the two- step clustering analysis. It should be noted that no Bonferroni correction was applied at this stage of initial analysis because the *t*-tests were only meant to identify which clinical characteristics were potentially relevant for phenotyping.

### Phenotyping

The two-step clustering analysis was run with the following criteria: AHI ≥ 15 (yes/no), atrial fibrillation (yes/no), hypertension (yes/no) and symptom severity ≥ 4.5 (yes/no). The analysis revealed five phenotypes and showed good cohesion and separation (average silhouette = 0.7), with AHI ≥ 15 as the most important input variable (importance = 1.00), followed by symptom severity (importance = 0.95), atrial fibrillation (importance = 0.88) and hypertension (importance = 0.79).

The five identified phenotypes were (Table 2):

- “High symptom severity”: 30 patients with high symptom severity, AHI < 15, no atrial fibrillation and, in eight cases, hypertension.
- “Low symptom severity”: 20 patients with low symptom severity, no atrial fibrillation, no hypertension and in most cases AHI ≥ 15.
- “Unclassified”: 28 patients, including all 21 patients with atrial fibrillation.
- “Hypertension”: 26 patients with hypertension, AHI ≥ 15, high symptom severity and no atrial fibrillation.
- “No comorbidity”: 47 patients without comorbidities (no atrial fibrillation, no hypertension), AHI ≥ 15 and high symptom severity.

**Table 2:** The five phenotypes of OSA patient groups and their characteristics

Phenotype	AHI ≥ 15										SS ≥ 4.5	
				Atrial fibrillation				Hypertension				
	yes	no		yes	no			yes	no		yes	no
No-comorbidity type (*n* = 47)	47	0		0	47			0	47		47	0
Hypertension type (*n* = 26)	26	0		0	26			26	0		26	0
High-symptom type (*n* = 30)	0	30		0	30			8	22		30	0
Low-symptom type (*n* = 20)	15	5		0	20			1	19		0	20
Unclassified type (*n* = 28)	26	2		21	7			20	6		11	17

Note. Overview of the five phenotypes specified by means of AHI ≥ 15, comorbidity and symptom severity (SS) ≥ 4.5. The number of patients is shown in each cell. AHI = Apnea- Hypopnea-Index; SS = symptom severity

### Differences between the five phenotypes

**Fig. 1:**
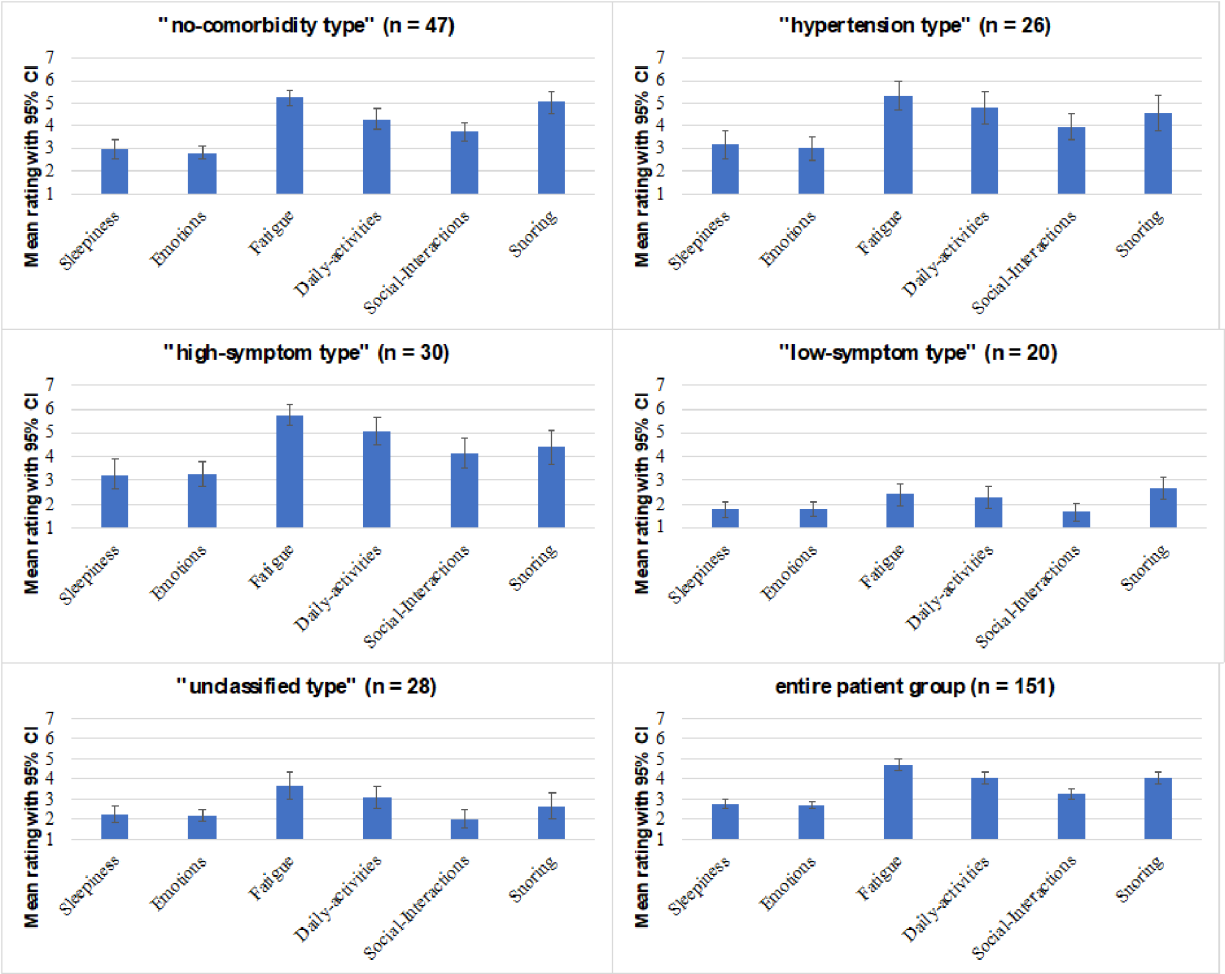
Representation of the mean ratings for the PRAQ scales

The mean PRAQ scores were calculated for all phenotypes and are presented in Fig. 1.

This figure shows the mean ratings for the PRAQ scales together with their 95% confidence intervals (CI), per phenotype and for the entire group of OSA patients. The inputs for the clusters were AHI ≥ 15 (yes/no), atrial fibrillation (yes/no), hypertension (yes/no) and symptom severity ≥ 4.5 (yes/no). The higher the score on the y-axis, the more severe the symptom is.

**Fig. 2:**
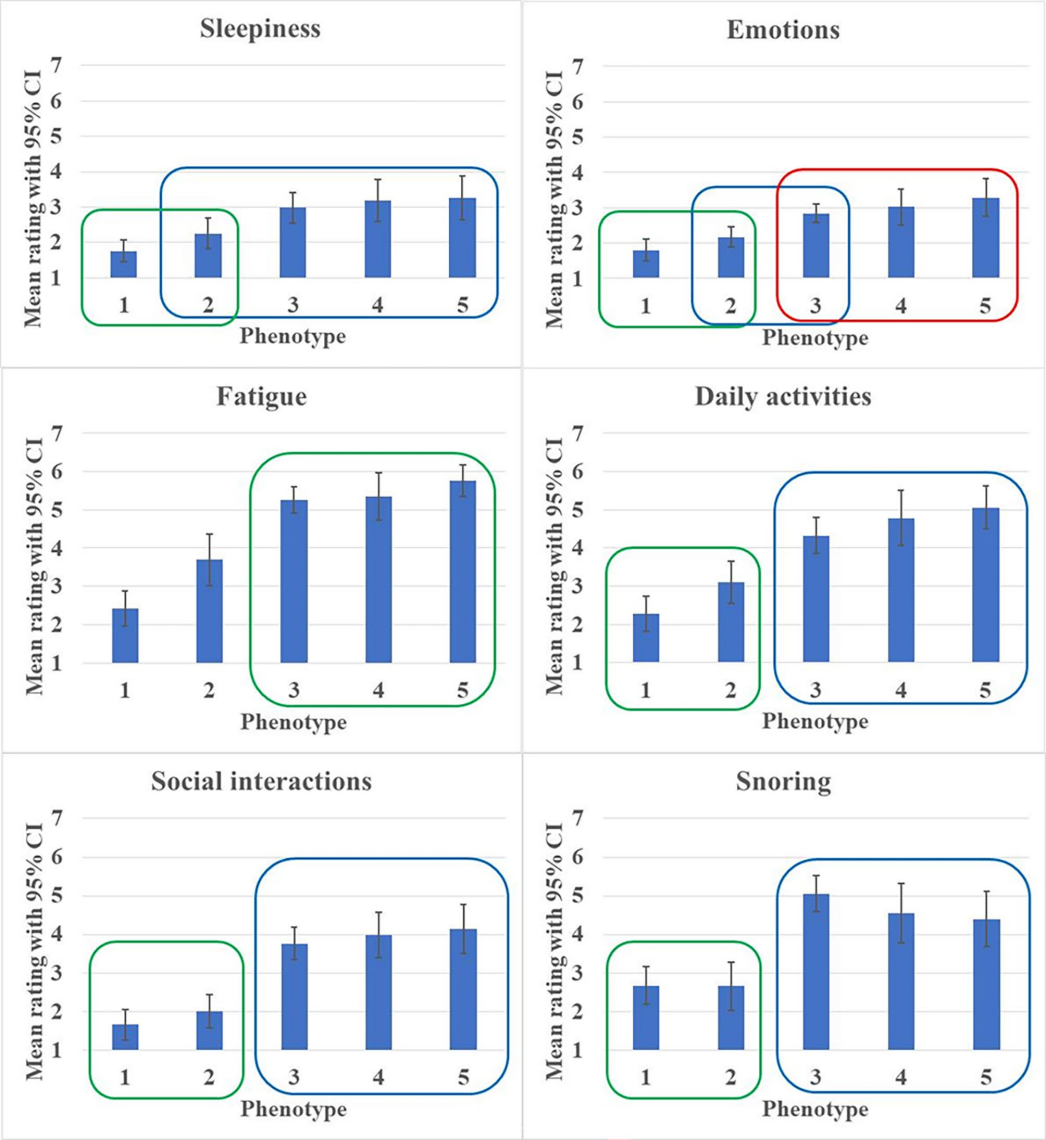
Representation of the mean ratings for the individual PRAQ scales

Multivariate ANOVA with the PRAQ scales and the ESS as dependent variables and phenotype as the fixed factor revealed significant differences between the mean PRAQ and ESS scores for the different phenotypes (all *p* <.001; except for ESS, for which *p* =.004). Subsequent post hoc analysis using the Tukey method revealed that the “low symptom severity” type experienced the least symptoms for all the PRAQ scales (see Fig. 2). The “hypertension”, “high symptom severity” and “no comorbidity” types experienced the most severe subjective symptoms and there were no significant differences between these three phenotypes. The “unclassified” type did not differ significantly from the “low symptom severity” type in experienced symptom severity, except for the “Fatigue” scale, for which the “low symptom severity” type scored significantly lower (*M* = 2.4, *SD* = 1.0) than the “unclassified” type (*M* = 3.7, *SD* = 1.8). All other phenotypes had mean values above 5.2 (which is above the severity level of 4.5).

This figure shows the mean ratings for the individual PRAQ scales together with their 95% confidence intervals (CI), as a function of phenotype. The phenotypes on the horizontal axis are predominantly ordered from low to high severity symptoms: 1 “low-symptom type”, 2 “unclassified type”, 3 “no-comorbidity type”, 4 “hypertension type”, and 5 “high-symptom type”. The Tukey post-hoc test showed that encircled phenotypes do not significantly differ from each other (with α = 0.05).

Figure 2 might suggest that there are only two phenotypes. Though, this is only true for half of the symptoms, i.e. for Daily activities, Social interaction and Snoring. For the other three symptoms (i.e., Sleepiness, Emotions, Fatigue) this is not true, based on statistical significance. Moreover, the comorbidity and AHI scores might also impact the extent to which the experienced symptom severity will improve after CPAP treatment. Therefore, the number of phenotypes cannot be reduced to only two.

## Discussion

### Statement of principal findings

To enable a more patient-oriented approach for the treatment of OSA patients, the purpose of this study was to phenotype OSA patients based on their symptom severity and clinical characteristics (AHI ≥ 15, atrial fibrillation and hypertension) by performing a two-step clustering analysis. We established five different phenotypes: “no comorbidity”, “hypertension”, “high symptom severity”, “low symptom severity” and “unclassified”.

### Strengths and limitations

This study, conducted in daily practice, has established a link between Patient Reported Outcome Measures (PROMS) and OSA phenotypes as a first step towards optimal personalized treatment. The findings should be seen as an indication for further research with a larger sample size to confirm these five phenotypes. Additionally, the outcomes should be interpreted in light of the study’s limitations.

First, it should be noted that not all reported symptoms can be fully attributed to OSA because patients might have other morbidities, such as depressive symptoms, that may also affect the perceived fatigue and sleep quality [23].

Second, as in previous studies [14, 15], female patients were underrepresented and therefore the findings on the differences on clinical characteristics in male and females should be interpreted with care. Because of the low sample size in women, which may inflate the risk of false positives and false negatives, we chose not to identify gender-specific symptoms or establish gender-specific phenotypes.

### Interpretation within the context of the wider literature

The importance of the clinical characteristics and symptom severity of patients was illustrated by establishing and comparing the phenotypes. Patients of “low symptom severity” type had relatively low levels of symptom severity, but 75% of these patients had AHI ≥ 15. This study also identified a “high symptom severity” type with a high levels of subjective symptom severity and AHI between 5 and 15. This might indicate that for these patients there is no significant relationship between the AHI and the severity of subjective symptoms, which confirms the results of previous studies [1, 2, 3]: AHI is needed but, as already known, the criterion AHI ≥ 15 alone is not sufficient to diagnose OSA and start CPAP treatment.

Both the “hypertension” and “no comorbidity” types had AHI ≥ 15 as well as high symptom severity, with respectively hypertension as a comorbidity or no comorbidity. According to the study of Sánchez-de-la-Torre et al., it is yet unknown whether patients with hypertension benefit from CPAP treatment, although for some individual cases improvements have been reported [24, 25]. It is important to assess whether both phenotypes benefit from CPAP treatment.

Patients in the “unclassified” type had the lowest levels of symptom severity, where it is known that patients with atrial fibrillation (included in the “unclassified” type) experience few OSA-related symptoms [2]. In this phenotype, CPAP treatment should focus less on reducing symptoms and more on improving the cardiac arrhythmia [26].

It is debatable whether the “low symptom severity” group should be treated, as it included only one patient with hypertension. Within this phenotype, 75% had AHI ≥ 15, while 25% did not present clinically relevant OSA or severe subjective symptoms. Their primary complaints were nightly symptoms such as snoring, feeling of suffocation, disturbed sleep and daytime sleepiness reported during the anamnesis. Although sleepiness is often assessed clinically, the ESS is not consistently recognized as a reliable measure [27, 28]. This suggests that standardized questionnaires and clinical criteria may not always capture the true severity of symptoms, which is essential for accurate diagnosis. Developing a new, shorter and more targeted questionnaire based on our phenotyping may help identify underlying problems more effectively during anamnesis.

The importance of gaining a more detailed picture of OSA patients was underlined by the finding that 44.4% of our patients was not obese, despite the typical description of an OSA patient as an overweight male. The STOP-BANG questionnaire, for instance, already results in an indication of OSA in a male with BMI > 35 and neck circumference > 40 cm, of which the latter appeared to be less predictive compared to the first two criteria [29]. Furthermore, in our study the average BMI for males was slightly lower than for females, as was the percentage of obese males versus obese females. Moreover, overweight patients did not experience other symptoms compared to patients who were not overweight. Therefore, we argue that, based on our study, the BMI may not be a useful classifier for OSA phenotyping.

The “Snoring” scale was established in this study and is relevant because patients are sometimes suspected of OSA based on their snoring behaviour (it is also one of the criteria in the STOP-BANG questionnaire) [29]. It must be noted that the OSA patient’s partner plays a crucial role in detecting the snoring and could be bothered by the snoring noise. We assume that the question about the snoring loudness is often scored by the patient’s partner rather than by the patient. The patient, in turn, could feel upset by others being disturbed by their snoring. Thus, although there is a commonality between the two snoring questions, they are reported from different perspectives and should both be maintained or at least included in a new OSA-specific questionnaire.

### Implications for policy, practice and research

Clinicians can use the results on the OSA phenotypes for personalized treatment selection (i.e., tailor CPAP treatment beyond AHI scores), refinement of diagnostic criteria (i.e., consider symptom severity alongside traditional measures), improved patient communication (i.e., explaining phenotypes to improve understanding of condition and treatment rationale), comprehensive patient assessment (e.g., involving partners regarding snoring-related symptoms). To identify the underlying problems of OSA patients we propose to use our modified PRAQ scales, and to facilitate a more patient-oriented prognosis and treatment, we propose to evaluate the treatment effectiveness based on the five reported phenotypes.

## Conclusion

This study aimed to phenotype a heterogeneous group of 151 OSA patients scheduled for CPAP treatment. We identified five phenotypes: (1) a “no comorbidity” type and (2) a “hypertension” type, both with AHI ≥ 15; (3) a “high symptom severity” type, with all patients having AHI < 15; (4) a “low symptom severity” type; and (5) an “unclassified” type that includes all patients with atrial fibrillation. The phenotypes primarily differ in snoring severity, symptoms affecting daily activities and experienced fatigue, but not in daytime sleepiness or ESS scores. This suggests that fatigue and snoring are key aspects to assess alongside clinical criteria. To better predict CPAP treatment effectiveness, we recommend evaluating these five phenotypes using our modified PRAQ scales.

## Data Availability

The data underlying this article will be shared on reasonable request to the corresponding author.

## Abbreviations

AHI: Apnea–hypopnea index
BMI: Body mass index
CI: Confidence intervals
CPAP: Continuous positive airway pressure
DM: Type II - Diabetes mellitus type 2
ESS: Epworth Sleepiness Scale
OSA: Obstructive sleep apnea
PRAQ: Patient Reported Apnea Questionnaire
PROMS: Patient Reported Outcome Measures
SS: Symptom severity
